# Molecular Docking and In-Silico Analysis of Natural Biomolecules against Dengue, Ebola, Zika, SARS-CoV-2 Variants of Concern and Monkeypox Virus

**DOI:** 10.3390/ijms231911131

**Published:** 2022-09-22

**Authors:** Mackingsley Kushan Dassanayake, Teng-Jin Khoo, Chien Hwa Chong, Patrick Di Martino

**Affiliations:** 1 School of Pharmacy, Faculty of Science and Engineering, University of Nottingham Malaysia, Jalan Broga, Semenyih 43500, Malaysia; 2 Department of Chemical and Environmental Engineering, Faculty of Science and Engineering, University of Nottingham Malaysia, Jalan Broga, Semenyih 43500, Malaysia; 3 BCMI Research Group, ERRMECe Laboratory, Cergy Paris University, 95000 Cergy-Pontoise, France

**Keywords:** molecular docking, dengue, Zika, Ebola, monkeypox, SARS-CoV-2, bacterial AMPs, biomolecules

## Abstract

The emergence and rapid evolution of human pathogenic viruses, combined with the difficulties in developing effective vaccines, underline the need to develop innovative broad-spectrum antiviral therapeutic agents. The present study aims to determine the *in silico* antiviral potential of six bacterial antimicrobial peptides (AMPs), two phytochemicals (silvestrol, andrographolide), and two bacterial secondary metabolites (lyngbyabellin A, hapalindole H) against dengue virus, Zika virus, Ebola virus, the major variants of SARS-CoV-2 and monkeypox virus. The comparison of docking scores obtained with natural biomolecules was performed with specific neutralizing antibodies (positive controls for ClusPro) and antiviral drugs (negative controls for Autodock Vina). Glycocin F was the only natural biomolecule tested to show high binding energies to all viral surface proteins and the corresponding viral cell receptors. Lactococcin G and plantaricin ASM1 also achieved high docking scores with all viral surface proteins and most corresponding cell surface receptors. Silvestrol, andrographolide, hapalindole H, and lyngbyabellin A showed variable docking scores depending on the viral surface proteins and cell receptors tested. Three glycocin F mutants with amino acid modifications showed an increase in their docking energy to the spike proteins of SARS-CoV-2 B.1.617.2 Indian variant, and of the SARS-CoV-2 P.1 Japan/Brazil variant, and the dengue DENV envelope protein. All mutant AMPs indicated a frequent occurrence of valine and proline amino acid rotamers. AMPs and glycocin F in particular are the most promising biomolecules for the development of broad-spectrum antiviral treatments targeting the attachment and entry of viruses into their target cell.

## 1. Introduction

The emergence and evolution of disease-causing viruses have posed a phenomenal threat to human health and have become a tremendous challenge to modern medicine and the global economy. These viruses are largely zoonotic in origin, i.e., they originate in a particular animal reservoir species and are transmitted to humans [1]. Depending on its potential to infect and transmit among humans, an emerging virus can result in a few sporadic cases, leading to a localized outbreak or can develop into an epidemic or even into a global pandemic in the worst scenarios. Such events of emergence over the past decades are numerous and varied in occurrence [2]. The management of these viral infections has become extremely challenging due to the ability of viruses to mutate and evolve over time under the influence of environmental, ecological and socio-economic factors, in particular, increasing globalization and climate change [2,3]. Examples of these outbreak-causing agents include the dengue virus, influenza viruses that cause swine and avian flu, emerging viruses such as Ebola, Zika, MERS, and SARS coronaviruses, and now the monkeypox virus [4,5]. Some of these viruses undergo high mutation rates and/or genome re-assortment, which allow antiviral drug resistance, host immune evasion, and reduced response to vaccines via antigenic shift and antigenic drift [6,7]. Viral interactions based on ecology, genetics, and host cell entry that determine the emergence of these viruses are extremely complex, making it impossible to predict the mechanism of the next epidemic or pandemic [8,9]. Thus, when a viral outbreak emerges and raises fears of a pandemic, a coordinated global response is needed that includes individual protection, social distancing, quarantines, information campaigns, and the development of antiviral treatments and vaccines [10,11]. Vaccine development takes time, but the development of antiviral treatments can be anticipated through the development of broad-spectrum drugs targeting a wide range of viruses [12,13,14,15].

The recent COVID-19 pandemic has shown that strategies to initiate the development of suitable innovative treatments for emerging viruses are crucial [16]. In recent years, studies that focus on natural products such as bioactive secondary metabolites and antimicrobial peptides (AMPs) from microorganisms and plants have been of great interest among researchers with the aspiration of identifying novel antiviral drugs against emerging viruses that cause epidemics and pandemics [17,18,19]. Recently emerging viruses that lead to pandemics include human immunodeficiency virus, SARS, MERS, hantavirus, dengue, West Nile virus, and Ebola and Zika virus [20]. Viral pathogens that caused major global pandemics include avian influenza A/H3N (Russian flu) during 1889–1893, avian influenza A/H1N1 (Spanish flu) during 1918–1919, avian influenza A/H2N2 (Asian flu) during 1957–1959, avian influenza A/H3N2 (Hong Kong flu) during 1968–1970, SARS-CoV disease between 2002–2003, swine flu caused by influenza A/H1N1, MERS-CoV disease 2015 and COVID-19 by SARS-CoV-2 at present [21]. Potential viral targets for these new antiviral drugs include capsid or envelope structural proteins [22,23]. Advances in computer technology permeate many aspects of drug discovery in the present day. Such technologies include virtual screening for hit identification and techniques for lead optimization which contribute towards low cost and safe screening of potential agents for the purpose of rational drug discovery. Virtual screening can be categorized into structure-based and ligand-based methods [24]. Molecular docking is the most commonly applied technique for virtual screening of molecular interactions since the early 1980s. Computer programs based on a variety of algorithms have been developed to perform molecular docking studies, as this virtual screening technique has become an increasingly important and critical tool in pharmaceutical research [25].

This study was conducted with a view to identifying new broad-spectrum natural antiviral agents targeting the attachment of the viral particle and its entry into the target cell. We herein report the binding potential of bioactive molecules naturally produced by bacteria and plants to surface viral proteins and corresponding cell receptors using molecular docking computer software in a virtual setting.

## 2. Results

The results of the *in silico* experiments reveal potential profound interactions between some biomolecule drug candidates and viral proteins or cellular virus receptors associated with dengue, Ebola, Zika, monkeypox viruses, and variants of SARS-CoV-2. The higher docking energy score was used to determine the docking strength of each ligand molecule with its respective receptor. The docking energy scores of reference material were used to compare the effectiveness of each biomolecule drug candidate and its antiviral potential. The data for molecular docking analyses for reference materials are summarized in Table 1. Using Autodock Vina software, the cut-off value for predicting high docking energy between a ligand and a receptor has been set at −6 kcal/mol in previous studies [26,27]. The ClusPro energy score reflects the attempt to achieve the native source with the lowest free binding energy [28]. The antiviral antibodies used as positive controls and cellular virus receptors indicated very high binding energy when docked with their corresponding surface viral proteins, with docking scores ranging from −749.6 to −1239.9 kcal/mol. Despite their mechanism of action targeting the replication machinery of the viral genome, the antiviral drugs Brincidofovir, Molnupiravir, Remdesevir, and Sofosbuvir showed docking scores with surface viral proteins slightly above the cut-off value described in the literature. The antiviral drug Tecovirimat inhibiting the envelope protein p37 which is essential for the extracellular transmission of the MPV also achieved a relatively low docking score for the viral protein A42R.

The comparison of docking scores obtained with natural biomolecules (AMPS, phytochemicals, or bacterial secondary metabolites) was performed with the negative and positive controls used in the study, i.e., specific neutralizing antibodies (positive controls for ClusPro) and antiviral drugs (negative controls for Autodock Vina). A docking score obtained with ClusPro that was greater than or equal to the results obtained with the positive control was considered high. A docking score obtained with Autodock Vina that was higher than the results obtained with the negative control was considered high.

Glycocin F showed high binding energies to all viral surface proteins and corresponding cell virus receptors tested (Table 2, Table 3, Table 4, Table 5, Table 6, Table 7 and Table 8). Plantaracin ASM1 and lactococcin G also showed high docking scores with all viral surface proteins tested and all cell receptors for DENV, ZIKV, EBOV, and SARS-VoV-2 variants, but not with monkeypox receptors (Table 3 and Table 4). The docking energy scores of bacterial AMPs ranged from −771 to −975.2 kcal/mol with DC-SIGN, from −793.5 to −1336.2 kcal/mL with AXL, from −756.8 to −1163.1 kcal/mol with TIM-1, from −609.3 to 1018.9 kcal/mol with ACE2, from −958.2 to −1505.5 kcal/mol with Toll-like receptor 5, from −719 to −1114.9 kcal/mol with CR3/Mac-1, and from −579.9 to −1134.2 kcal/mol with CD36 (Table 2). The docking energy scores of bacterial AMPs with spike (S) proteins of SARS-CoV-2 ranged as follows; for bacteriocin plantaricin ASM1 the range was −1237.5 to −1399.3 kcal/mol (Table 3), for bacteriocin lactococcin G the range was −1009.9 to −1262.3 kcal/mol (Table 4), for nisin the range was −741.3 to −826 kcal/mol (Table 5), for bacteriocin glycocin F the range was −1219.4 to −1756.7 kcal/mol (Table 6), for gardimycin the range was −938.1 to −998.4 kcal/mol (Table 7) and for surfactin the range was −884.5 to −975 kcal/mol (Table 8). Docking scores for DENV envelope protein, ZIKV protein E, EBOV glycoprotein, and A42R Profilin-like protein ranged from −990.5 to −1167.4 kcal/mol with bacteriocin plantaricin ASM1 (Table 3), ranged from −880.6 to −1056.6 kcal/mol with bacteriocin lactococcin G (Table 4), from −633.2 to −783.6 kcal/mol with nisin (Table 5), from −1009 to −1208.2 kcal/mol with bacteriocin glycocin F (Table 6), from −754 to 1009 kcal/mol with gardimycin (Table 7), and from −742 to −952.4 kcal/mol with surfactin (Table 8). Bacteriocin glycocin F exhibited the highest docking energy for the spike (S) protein of SARS-CoV-2 B.1.427 USA variant, with a docking score of −1756.7 kcal/mol (Table 6). Meanwhile, bacteriocin plantaricin ASM1 showed the highest docking energy for spike (S) protein of SARS-CoV-2 B.1.351 South African variant with a docking score of −1399.3 kcal/mol (Table 3). The highest docking energy for the Ebola virus glycoprotein was recorded with bacteriocin glycocin F, which indicated a docking score of −1208.2 kcal/mol. The interaction of specific amino acid residues of the bacterial AMPs and the surface viral proteins are also presented in Table 3, Table 4, Table 5, Table 6, Table 7 and Table 8. PyMOL indicated the presence of a variety of interacting amino acid residues for each docked molecule.

The two phytochemicals silvestrol and andrographolide, as well as the two bacterial secondary metabolites hapalindole H and lyngbyabellin A showed variable docking scores depending on the viral surface proteins tested (Table 9, Table 10, Table 11 and Table 12). Hapalindole H showed high docking scores with a wide panel of viral proteins from ZIKV, EBOV, SARS-CoV-2 B.1.1.7 UK, SARS-CoV-2 B.1.351 South African, SARS-CoV-2 P.1 Japan/Brazil and SARS-CoV-2 B.1.1.529 Omicron variants. Silvestrol showed high docking scores with the viral proteins of ZIKV, SARS-CoV-2 B.1.1.7 UK, SARS-CoV-2 B.1.351 South African, SARS-CoV-2 P.1 Japan/Brazil and SARS-CoV-2 B.1.1.529 Omicron variants of concern (Table 9). Andrographolide showed high docking energies with the viral proteins of EBOV, SARS-CoV-2 B.1.1.7 UK, and SARS-CoV-2 P.1 Japan/Brazil variants (Table 10). Lyngbyabellin A showed high docking scores only with SARS-CoV-2B.1.1.7 UK, SARS-CoV-2 P.1 Japan/Brazil, SARS-CoV-2 B.1.617.2 Indian, and SARS-CoV-2 B.1.1.529 Omicron variants (Table 11). Silvestrol recorded the highest docking energy of −8.4 kcal/mol for the spike protein of the SARS-CoV-2 B.1.351 South African variant (Table 9). Andrographolide showed the highest docking energy for the spike proteins of the SARS-CoV-2 B.1.1.7 UK variant with a value of −7.7 kcal/mol (Table 10). Lyngbyabellin A recorded the highest docking score of −9.0 kcal/mol for the spike protein of the SARS-CoV-2 B.1.1.7 UK variant (Table 11). Hapalindole H had the same highest docking energy for the spike proteins of the SARS-CoV-2 B.1.351 South African and SARS-CoV-2 P.1 Japan/Brazil variants with a value of −8.2 kcal/mol (Table 12). Silvestrol, lyngbyabellin A, andrographolide, and hapalindole H showed high docking scores to the DENV cellular receptor, but low docking energies to the ZIKV, MPV, and SARS-CoV-2 cellular receptors (Table 2). Hapalindole H but not silvestrol, lyngbyabellin A, and andrographolide showed high docking energy to the EBOV cellular receptor (Table 2).

**Figure 1:**
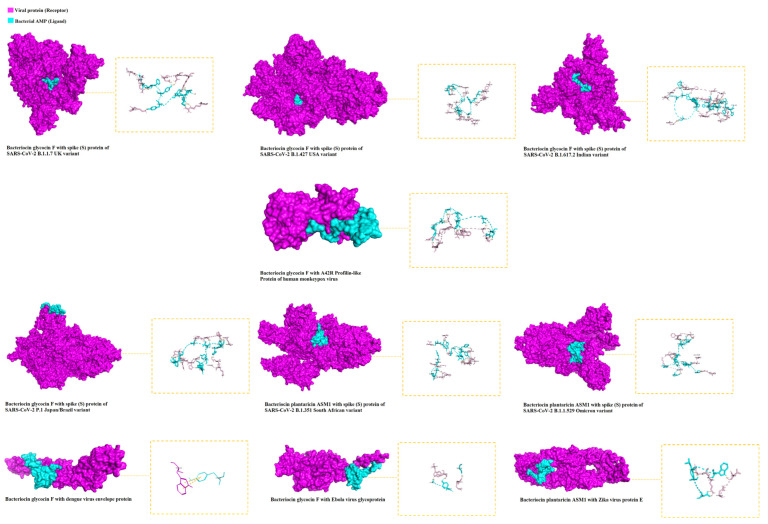
The 3D visualization of the molecular docking of bacterial AMPs with viral surface proteins and their corresponding amino acid residues (highest binding affinities between AMPs and each viral protein were selected). The viral protein (receptor) is illustrated in purple and the bacterial AMP (ligand) is illustrated in blue.

**Figure 2:**
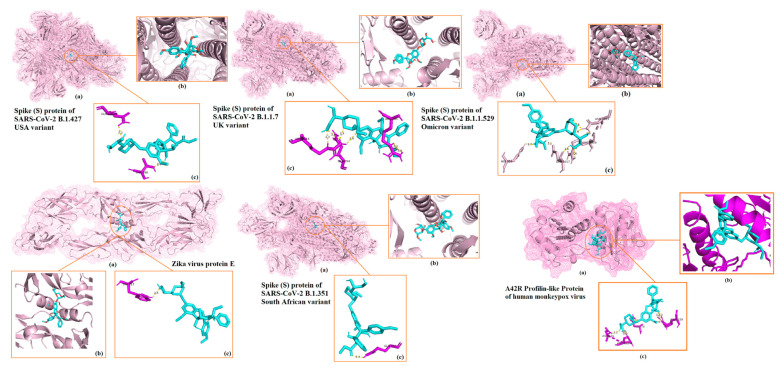
Results of the docking analysis showing the 6 main binding sites of silvestrol with the different viral surface proteins. The molecular interactions in the 3D structures were visualized in PyMOL. (**a**) Indicates binding domain. (**b**) Binding site. (**c**) Interacting amino acids of receptor (purple) and ligand (blue).

**Figure 3:**
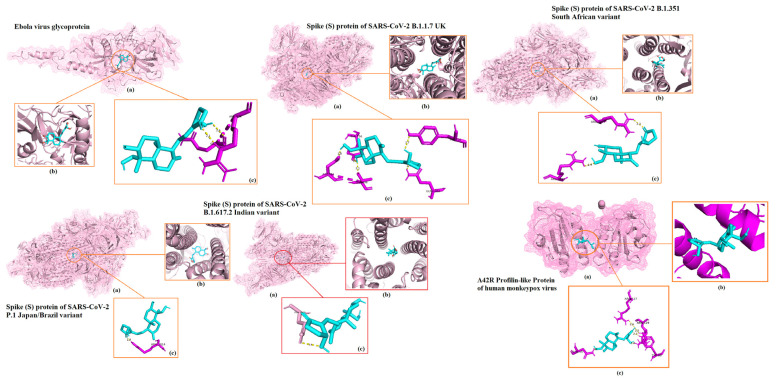
Results of the docking analysis showing the 6main binding sites of andrographolide with the different viral surface proteins. The molecular interactions in the 3D structures were visualized in PyMOL. (**a**) Indicates binding domain. (**b**) Binding site. (**c**) Interacting amino acids of receptor (purple) and ligand (blue).

**Figure 4:**
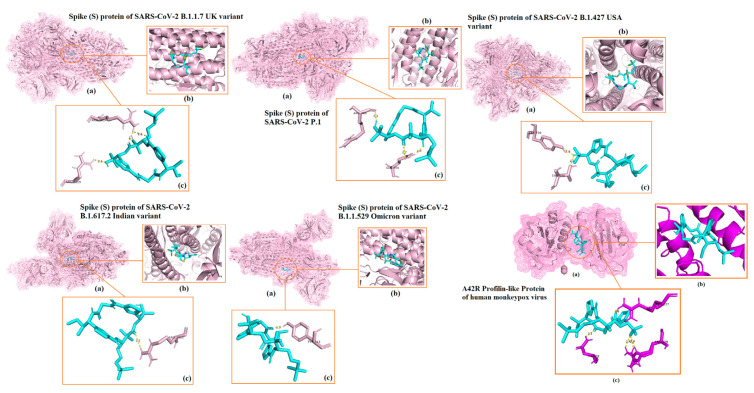
Results of the docking analysis showing the 6 main binding sites of lyngbyabellin A with the different viral surface proteins. The molecular interactions in the 3D structures were visualized in PyMOL. (**a**) Indicates binding domain. (**b**) Binding site. (**c**) Interacting amino acids of receptor (purple) and ligand (blue).

**Figure 5:**
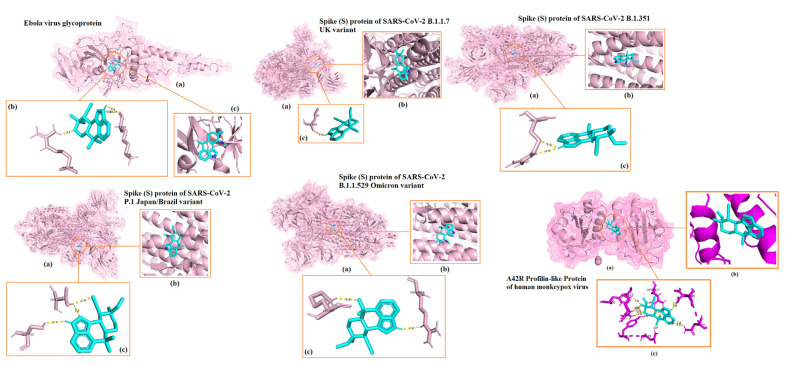
Results of the docking analysis showing the 6 main binding sites of hapalindole H with the different viral surface proteins. The molecular interactions in the 3D structures were visualized in PyMOL. (**a**) Indicates binding domain. (**b**) Binding site. (**c**) Interacting amino acids of receptor (purple) and ligand (blue).

**Figure 6:**
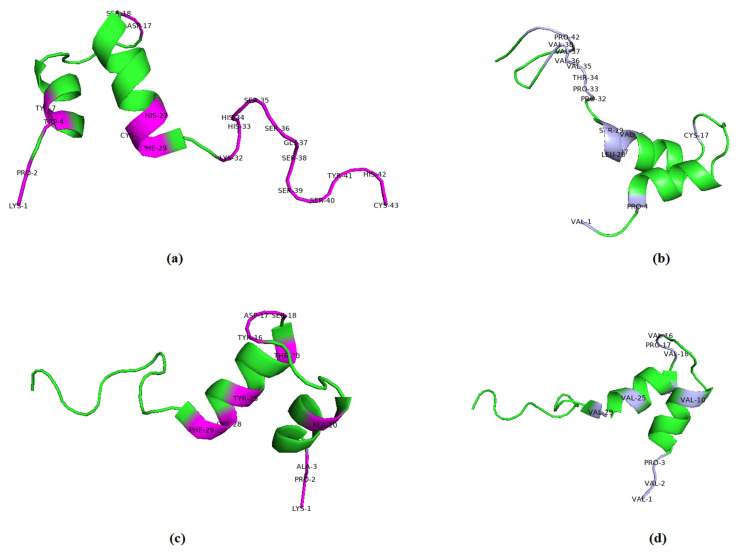
Comparison of wild-type and mutant bacteriocin glycocin F. (**a**) Wild-type bacteriocin glycocin F against the spike (S) protein of SARS-CoV-2 B.1.617.2 Indian variant. (**b**) Mutant bacteriocin glycocin F against the spike (S) protein of SARS-CoV-2 B.1.617.2 Indian variant. (**c**) Wild-type bacteriocin glycocin F against the spike (S) protein of against SARS-CoV-2 P.1 Japan/Brazil variant. (**d**) Mutant bacteriocin glycocin F against the spike (S) protein of SARS-CoV-2 P.1 Japan/Brazil variant. Amino acid residues of the wild-type protein appear in purple, whereas rotamers of amino acid residues of mutant protein appear in gray.

The 3D representations of the molecular docking results with the highest docking scores for each viral protein are shown in Figure 1. Best binding energy illustrations for the six targets for silvestrol, andrographolide, lyngbyabellin A, and hapalindole H with the surface viral proteins and interacting amino acid residues are illustrated in Figure 2, Figure 3, Figure 4 and Figure 5, respectively. The AMPs with the highest binding energies for the viral proteins were mutated and the mutants were then evaluated for their affinity for the viral proteins. Among the mutant AMPs tested, only three glycocin F mutants showed an increase in their docking energy with the spike protein of SARS-CoV-2 B.1.617.2 Indian variant, the spike protein of SARS-CoV-2 P.1 Japan/Brazil variant, and the DENV envelope protein compared to the corresponding wild-type AMPs, indicating improved molecular docking stability (Table 13 and Figure 6). This was not the case for the bacteriocin plantaricin ASM1 against the Zika virus protein E and against the spike (S) protein of SARS-CoV-2 B.1.351 South African variant, nor was it the case for the bacteriocin glycocin F against the spike (S) protein of SARS-CoV-2 P.1 Japan/Brazil variant (Table 13 and Figure 6). The frequent occurrence of amino acid rotamers, valine (VAL), and proline (PRO) has been detected in all mutant bacterial AMPs.

## 3. Discussion

The multitude of emerging viruses such as dengue, Zika, Ebola, monkeypox, and the recent occurrence of the COVID-19 pandemic show that the current antiviral therapeutic arsenal is not sufficient [2,4]. Vaccine development is costly and time-consuming, and large-scale administration can be difficult [29]. Furthermore, the development of antiviral treatments is hampered by the high evolutionary power of certain viruses such as coronaviruses or the Ebola virus. All this highlights the need to develop new broad-spectrum antiviral molecules, i.e., active against a wide range of viruses [30,31,32]. Ideally, these broad-spectrum antiviral treatments should be able to be active against new, totally unknown viruses or emerging viruses that are mutants or variants of known viruses.

**Figure 7:**
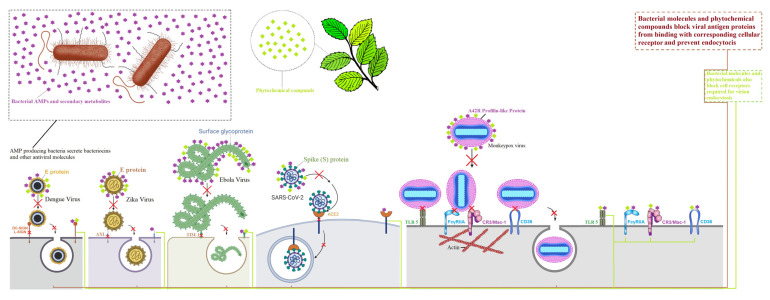
Proposed antiviral mechanism of action of bacterial molecules and phytochemicals against DENV, EBOV, ZIKV, MPV, and SARS-CoV-2, by targeting the interaction between viral surface proteins and cell receptors (DC-SIGN, AXL, TIM-1, ACE2, Toll-like receptor 5, FcγRIIA, CR3/Mac-1 and CD36). Phytochemicals are incapable of binding with AXL and ACE2 cell receptors and lyngbyabellin A is unable to bind with the surface E protein of DENV. These natural biomolecules may contribute to reducing viral pathogenesis in host cells.

Dengue, Ebola, Zika, and SARS-CoV-2 viruses use their structural surface proteins to attach and induce entry into host cells and initiate pathogenesis. Therefore, a therapeutic agent capable of inhibiting the attachment of these viruses to their corresponding host cell receptor would block the initiation of viral replication and early infection [33,34]. Viral surface proteins that bind to the surface receptor of the target cell are good candidates for vaccine development because they stimulate the production of blocking antibodies [35]. Various natural biomolecules are capable of blocking in vitro viral infection of a given virus type, usually by blocking the action of the replication machinery of the viral genome [36,37]. The present study was based on the assumption that blocking viral surface proteins and/or their corresponding cell receptors could also be achieved with natural biomolecules. We selected various natural biomolecules of bacterial or plant origin for which data in the literature have shown antiviral properties against different viruses. We tested *in silico* their ability to interact with proteins involved in viral recognition of cell surface receptors of the main emerging viruses. Thus, we targeted viral envelope proteins of dengue, Ebola, Zika, and SARS-CoV-2 viruses and corresponding cell surface receptors, in search of broad-spectrum antiviral molecules. Given its recent emergence in various countries, we also looked at the monkeypox virus. We relied on the few data published and/or accessible in databases to select the viral protein A42R and the cellular receptors Toll-like receptor 5, CR3/Mac-1, CD36, and FcγRIIA. Figure 7 illustrates the proposed mode of antiviral action of these biomolecules, which consists of blocking either the viral surface proteins or the corresponding cell receptors, or both.

Silvestrol, a secondary metabolite derived from *Aglaia* spp. showed broad-spectrum antiviral potential in the *in silico* study conducted as it was predicted to interact with viral surface proteins of two viral families (ZIKV, and some SARS-CoV-2 variants). However, binding predictions only showed a high docking score with DENV cellular receptor for silvestrol. Thus, silvestrol could block the interaction of DENV with target cells by binding to the cell receptor. For the ZIKV, and SARS-CoV-2 viruses, silvestrol would block solely viral surface proteins. Other mechanisms for the antiviral action of silvestrol have been previously reported in the literature [38,39,40]. A study conducted by Müller et al. showed that silvestrol inhibits the replication of HCoV-229E and MERS-CoV by suspending cap-dependent viral mRNA translation [41]. Various studies have shown that silvestrol can inhibit the eIF4A-dependent translation of viral mRNA of EBOV, ZIKV and hepatitis E virus [42,43]. A study by Henss et al. also showed that silvestrol can delay protein synthesis of the chikungunya virus and reduce viral RNA replication [44]. A recent investigation showed that silvestrol at a concentration of 10 nM reduces viral titers of SARS-CoV-2 up to 100-fold in infected human bronchial epithelial cells [45]. 

The phytochemical compound andrographolide, derived from *A. paniculate*, also showed broad-spectrum antiviral potential in the present *in silico* study. As with silvestrol, high docking scores were obtained when andrographolide was tested against the cellular receptors of DENV. Binding predictions also showed strong docking scores with the surface protein of EBOV and two variants of SARS-CoV-2. Thus, andrographolide could block the interaction of DENV with target cells by interacting with the corresponding cellular receptors. For EBOV and SARS-CoV-2, andrographolide would block solely viral surface proteins. Data from the literature have shown that andrographolide has various antiviral properties [46]. Andrographolide inhibits the replication of DENV and reduces infection in human HepG2 and HeLa cells [47]. Ethanol extracts of *A. paniculate* containing andrographolide inhibit the activity of the simian retrovirus in human A549 cells [48]. Andrographolide reduces CHIKV infection in human HepG2 cells by interfering with viral protein synthesis [49]. Andrographolide inhibits the expression of viral enveloped glycoproteins C and D of herpes simplex virus type 1 [50]. Andrographolide inhibits the activity of DENV and ZIKV [51]. Andrographolide decreases the viral load of SARS-CoV-2 in human Calu-3 cells [52] and is thought to inhibit the main protease of SARS-CoV-2 [53,54].

Secondary metabolites isolated from cyanobacteria are known for their potential antiviral activity against viral pathogens such as HIV, measles virus, adenovirus, influenza, herpes simplex virus, and Coxsackie [55,56,57]. A recent *in silico* study conducted by Aminu et al. showed that an indole alkaloid compound known as hapalindole derived from marine cyanobacteria has strong docking energy with SARS-CoV-2 spike protein [58]. In our study, hapalindole was found to have a high docking energy not only to surface proteins of different variants of SARS-CoV-2 but also to those of ZIKV and EBOV. Binding predictions also showed strong docking scores with the cellular receptors of DENV and EBOV for hapalindole. Thus hapalindole may have antiviral properties against EBOV by blocking its envelope glycoprotein and the corresponding receptor, antiviral properties against SRARS-CoV-2 and ZIKV by blocking only viral envelope proteins and DENV by blocking the cell receptor.

Glycocin F was the only AMP, and more broadly, the only natural biomolecule tested to show high binding energies to all viral surface proteins and corresponding cell virus receptors. Lactococcin G and plantaricin ASM1 also showed promising broad-spectrum *in silico* antiviral potential by targeting all the surface viral proteins and most of the corresponding cell surface receptors. The binding affinities obtained between these three AMPs and the corresponding viral surface proteins or cell receptors were very high (from −825.2 to −1756.7 kcal/mol). Among the AMPs tested, the lantibiotic bacteriocin produced by *L. lactis* known as nisin, is the only FDA-approved microbial-derived AMP up to date [59,60]. The antiviral activity of nisin against the bovine viral diarrhea virus (BVDV) has already been demonstrated [61]. An *in silico* study conducted by Balmeh et al. showed that bacteriocin glycocin F derived from *L. lactis* and bacteriocin plantaricin ASM1 derived from *L*. *plantarum* have high docking energy with SARS-CoV-23CL protease, RNA-dependent RNA polymerase RdRp and spike (S) envelope protein [19]. Surfactin is a powerful natural antimicrobial derived from *B*. *subtilis*, which suppresses the proliferation of porcine epidemic diarrhea virus (PEDV) and transmissible swine gastroenteritis virus (TGEV) in epithelial cells by inhibiting viral membrane fusion with host cells at concentrations between 15–50 μg/mL [62]. This mechanism of action is consistent with the *in silico* results presented here showing that gardimycin, glycocin F, lactococcin G, and plantaricin ASM1 may interact with the surface proteins of various viruses and different variants of the SARS-COV-2 virus, and with their corresponding cell surface receptors. This could explain recently published results showing that AMPs produced by probiotic strains of *Lactobacillus acidophilus* reduce symptoms of hospitalized patients with COVID-19 and improve antibody production against SARS-CoV-2 [60,61,63,64].

## 4. Methods

### 4.1. Ligands and Receptors Executed

#### 4.1.1. Ligands

A total of 19 ligands, including 9 reference molecules (4 antibodies and 5 antiviral drugs), 6 AMPs of bacterial origin, 2 phytochemicals, and 2 cyanobacterial secondary metabolites, were selected for the molecular docking experiments (Table S1). The antibodies and cellular virus receptors were used as positive controls for interaction with corresponding viral surface proteins. Antiviral drugs targeting enzymes involved in viral genome replication were used as negative controls for interaction with viral surface proteins. The three-dimensional (3D) structures of the selected macromolecules were downloaded from the Research Collaboratory for Structural Bioinformatics Protein (RCSB) PDB database (https://www.rcsb.org/) accessed on 10 August 2021 and from the National Library of Medicine (NLM) PubChem PDB database (https://pubchem.ncbi.nlm.nih.gov) accessed on 10 August 2021 in SDF format.

#### 4.1.2. Receptors

A total of 10 viral structural surface proteins from dengue, Ebola, Zika, SARS-CoV-2, and monkeypox viruses and 8 cellular virus receptors were selected as receptors for docking with the designated ligands. The viral targets were the dengue virus envelope (E) protein (PDB ID: 1TG8), the Ebola virus surface glycoprotein (PDB ID: 5JQ3), the Zika virus envelope protein E (PDB ID: 5JHM), the SARS-CoV-2 spike (S) proteins of the UK variant B.1.1.7 (PDB ID: 7LWS), the South African variant.1.351 (PDB ID: 7LYK), the Japan/Brazil variant P.1 (PDB ID: 7M8K), the USA variant B.1.427 (PDB ID: 7N8H), the Indian variant B.1.617.2 (PDB ID:7V7O), the Omicron variant B.1.1.529 (PDB ID: 7T9J) and the A42R Profilin-like protein of monkeypox virus (PDB ID: 4QWO). Cellular receptors for the corresponding viral protein include DC-SIGN (PDB ID: 1SL4) for the dengue virus envelope protein, AXL (PDB ID: 5U6B) for the Zika virus protein E, TIM-1 (PDB ID: 2OR) for Ebola virus glycoprotein, Toll-like receptor 5 (PDB ID: 3J0A), CR3/Mac-1 (PDB ID: 4M76), CD36 (PDB ID: 5LGD), and FcγRIIA (PDB ID: 1H9V) for A42R Profilin-like protein of MPV, and ACE2 (PDB ID: 1R42) for spike (S) protein of SARS-CoV-2.

### 4.2. Modeling and Preparation of Selected Macromolecules

The PDB structures of viral proteins and phytochemical compounds were modified using AutoDockTools (version 1.5.7, La Jolla, CA, USA), where water molecules were discarded and hydrogen bonds and Kollman charges were added. The modified SDF and PDB files were converted to Protein Data Bank, Partial Charge (Q), and Atom Type (T) (PDBQT) format before being analyzed to optimize docking efficiency.

### 4.3. Molecular Docking Analysis

The molecular docking of smaller macromolecules such as phytochemical compounds with the selected viral structural proteins was performed by using AutoDock Vina (version 1.1.2, La Jolla, CA, USA). The docking was performed at a default grid box dimension of 40 Å × 40 Å × 40 Å and the energy range was set at 4 and exhaustiveness was set at 8 [65]. In the case of larger macromolecules, i.e., bacterial AMPs and reference antibodies, molecular docking with the selected viral structural proteins was performed by the application of the ClusPro 2.0 supercomputer-based online server [66,67,68,69].

### 4.4. Estimation of Binding Free Energy/Docking Energy and Determination of the Root Mean Square Distance

AutoDock Vina software and ClusPro online server were used to calculate and estimate the docking energies/binding free energies of ligand–receptor complexes in kcal/mol. The balanced output of model rank 0 result of ClusPro was selected as the most accurate output. Whereas, the Autodock Vina log file of the docked molecule indicating the binding affinity allocated to a root mean square distance (RMSD) value of zero was selected as the best result.

### 4.5. Simulation of Molecular Ligand-Receptor Interactions

Simulation and visualization of molecular interactions indicating the active binding sites of ligand-receptor complexes and their amino acid sequences were performed using PyMOL (version 2.5.2) molecular visualization system [70]. 

### 4.6. Mutation and Structure Modeling of Bacterial AMPs

The two AMPs bacteriocin glycocin F and plantaricin ASM1, which showed the highest docking scores with certain viral surface proteins, were subjected to mutation by initiating the mutagenesis feature of the PyMOL software. Mutagenesis was performed in order to determine the possibility of further increasing the docking strength of bacterial AMPs. The basis of PyMOL mutagenesis involves the replacement of amino acid residues of AMPs with their corresponding rotamers that denote the highest percentage of mutation probability. Mutant AMPs remodeled as ligands were re-docked using ClusPro with their corresponding viral protein receptors to determine the increase in binding energies induced by the effects of the mutations. Interaction figures of the remodeled peptides of the mutant AMPs were designed and illustrated by PyMOL software [19,70].

## 5. Conclusions

In conclusion, glycocin F is the natural biomolecule with the highest potential to develop a broad-spectrum antiviral agent. Remarkably, the binding energies of glycocin F for surface viral proteins are at least as high as those of cell virus receptors. This highlights the interest in further studying the antiviral activity of these natural molecules to develop broad-spectrum antiviral agents. The interaction of glycocin F with different viral surface proteins and their cell receptors should now be demonstrated experimentally. As indicated by the *in silico* mutagenesis results, the sequence of this antimicrobial peptide can certainly be optimized, this will also have to be developed experimentally.

## Figures and Tables

**Table 1:** Molecular docking data of reference materials with selected viral proteins.

Ligand (Negative Control)		Receptor		Docking Energy/Binding Affinity (kcal/mol)
Molecule ID	Name	PDB ID	Name	
2R69	Fab 1A1D-2 (DENV neutralizing antibody)	1TG8	Dengue virus envelope protein	−803
5JHL	2A10G6 Fab (ZIKV neutralizing antibody)	5JHM	Zika virus protein E	−859.5
5FHB	mAb100 (EBOV neutralizing antibody)	5JQ3	Ebola virus glycoprotein	−954.1
7JMX	COVA1-16 Fab (SARS-CoV-2 neutralizing antibody)	7LWS	Spike (S) protein of SARS-CoV-2 B.1.1.7 UK variant	−801.9
		7LYK	Spike (S) protein of SARS-CoV-2 B.1.351 South African variant	−789.2
		7M8K	Spike (S) protein of SARS-CoV-2 P.1 Japan/Brazil variant	−850.7
		7N8H	Spike (S) protein of SARS-CoV-2 B.1.427 USA variant	−806
		7V7O	Spike (S) protein of SARS-CoV-2 B.1.617.2 Indian variant	−874.9
		7T9J	Spike (S) protein of SARS-CoV-2 B.1.1.529 Omicron variant	−754.3
145996610	Molnupiravir (anti-SARS-CoV-2 drug candidate)	7LWS	Spike (S) protein of SARS-CoV-2 B.1.1.7 UK variant	−7.2
		7LYK	Spike (S) protein of SARS-CoV-2 B.1.351 South African variant	−7.5
		7M8K	Spike (S) protein of SARS-CoV-2 P.1 Japan/Brazil variant	−7.2
		7N8H	Spike (S) protein of SARS-CoV-2 B.1.427 USA variant	−8.3
		7V7O	Spike (S) protein of SARS-CoV-2 B.1.617.2 Indian variant	−7.8
		7T9J	Spike (S) protein of SARS-CoV-2 B.1.1.529 Omicron variant	−7.7
121304016	Remdesivir (anti-EBOV drug candidate)	5JQ3	Ebola virus glycoprotein	−7.5
45375808	Sofosbuvir (anti-DENV and anti-ZIKV drug candidate)	1TG8	Dengue virus envelope protein	−6.5
		5JHM	Zika virus protein E	−7.1
483477	Brincidofovir (antiviral drug for MPV)	4QWO	A42R Profilin-like protein of MPV	−9.8
16124688	Tecovirimat (antiviral drug for MPV)			−9.6
1SL4	DC-SIGN	1TG8	Dengue virus envelope protein	−879.7
5U6B	AXL	5JHM	Zika virus protein E	−986.4
2OR8	TIM-1	5JQ3	Ebola virus glycoprotein	−1012.2
3J0A	Toll-like receptor 5	4QWO	A42R Profilin-like protein of MPV	−1239.9
4M76	CR3/Mac-1			−937.6
5LGD	CD36			−941.5
1H9V	FcγRIIA			−749.6
1R42	ACE2	7LWS	Spike (S) protein of SARS-CoV-2 B.1.1.7 UK variant	−942.7
		7LYK	Spike (S) protein of SARS-CoV-2 B.1.351 South African variant	−1152.8
		7M8K	Spike (S) protein of SARS-CoV-2 P.1 Japan/Brazil variant	−1003.5
		7N8H	Spike (S) protein of SARS-CoV-2 B.1.427 USA variant	−903
		7V7O	Spike (S) protein of SARS-CoV-2 B.1.617.2 Indian variant	−1096
		7T9J	Spike (S) protein of SARS-CoV-2 B.1.1.529 Omicron variant	−969.9

SARS-CoV-2: Severe acute respiratory syndrome coronavirus 2; DENV: Dengue virus; EBOV: Ebola virus; ZIKV: Zika virus.

**Table 2:** Molecular docking data of cellular virus receptors with selected ligands.

Receptor (Positive Control)		Ligand		Docking Energy/Binding Affinity (kcal/mol)
PDB ID	Name	Molecule ID	Name	
1SL4	DC-SIGN	2MVI	Bacteriocin plantaricin ASM1	−928
		2KUY	Bacteriocin glycocin F	−975.2
		2JPK	Bacteriocin lactococcin-G	−964.5
		5XHB	Nisin	−816.5
		1AJ1	Gardimycin	−821.3
		1JMK	Surfactin	−771
		11787114	Silvestrol	−6.9
		5318517	Andrographolide	−7.9
		10032587	Lyngbyabellin A	−6.7
		21671525	Hapalindole H	−7.9
5U6B	AXL	2MVI	Bacteriocin plantaricin ASM1	−1336.2
		2KUY	Bacteriocin glycocin F	−1225.2
		2JPK	Bacteriocin lactococcin-G	−1071.4
		5XHB	Nisin	−793.5
		1AJ1	Gardimycin	−892.5
		11787114	Silvestrol	−3.6
		5318517	Andrographolide	−3
		10032587	Lyngbyabellin A	−2.8
		21671525	Hapalindole H	−3.7
		1JMK	Surfactin	−806.3
2OR8	TIM-1	2MVI	Bacteriocin plantaricin ASM1	−1142.2
		2KUY	Bacteriocin glycocin F	−1163.1
		2JPK	Bacteriocin lactococcin-G	−959.7
		5XHB	Nisin	−756.8
		1AJ1	Gardimycin	−812.1
		1JMK	Surfactin	−798.6
		11787114	Silvestrol	−3.6
		5318517	Andrographolide	−7.4
		10032587	Lyngbyabellin A	−7.1
		21671525	Hapalindole H	−8.3
7JMX	ACE2	2MVI	Bacteriocin plantaricin ASM1	−996.5
		2KUY	Bacteriocin glycocin F	−985.2
		2JPK	Bacteriocin lactococcin-G	−1018.9
		5XHB	Nisin	−609.3
		1AJ1	Gardimycin	−868.4
		1JMK	Surfactin	−697
		11787114	Silvestrol	−4.0
		5318517	Andrographolide	−3.4
		10032587	Lyngbyabellin A	−3.2
		21671525	Hapalindole H	−2.8
3J0A	Toll-like receptor 5	2MVI	Bacteriocin plantaricin ASM1	−1372.7
		2KUY	Bacteriocin glycocin F	−1505.5
		2JPK	Bacteriocin lactococcin-G	−1331.7
		5XHB	Nisin	−958.2
		1AJ1	Gardimycin	−1060.3
		1JMK	Surfactin	−1126.3
		11787114	Silvestrol	−6.8
		5318517	Andrographolide	−6.3
		10032587	Lyngbyabellin A	−6.4
		21671525	Hapalindole H	−7.1
4M76	CR3/Mac-1	2MVI	Bacteriocin plantaricin ASM1	−1114.9
		2KUY	Bacteriocin glycocin F	−1068.0
		2JPK	Bacteriocin lactococcin-G	−825.2
		5XHB	Nisin	−719.0
		1AJ1	Gardimycin	−815.1
		1JMK	Surfactin	−869.7
		11787114	Silvestrol	−7.3
		5318517	Andrographolide	−7.6
		10032587	Lyngbyabellin A	−6.1
		21671525	Hapalindole H	−6.8
5LGD	CD36	2MVI	Bacteriocin plantaricin ASM1	−910.8
		2KUY	Bacteriocin glycocin F	−1134.2
		2JPK	Bacteriocin lactococcin-G	−906.6
		5XHB	Nisin	−579.9
		1AJ1	Gardimycin	−771.7
		1JMK	Surfactin	−711.7
		11787114	Silvestrol	−7.7
		5318517	Andrographolide	−7.7
		10032587	Lyngbyabellin A	−6.8
		21671525	Hapalindole H	−8.8
1H9V	FcγRIIA	2MVI	Bacteriocin plantaricin ASM1	−886.3
		2KUY	Bacteriocin glycocin F	−995.1
		2JPK	Bacteriocin lactococcin-G	−986.6
		5XHB	Nisin	−606.6
		1AJ1	Gardimycin	−762.7
		1JMK	Surfactin	−706.5
		11787114	Silvestrol	−6.6
		5318517	Andrographolide	−6.8
		10032587	Lyngbyabellin A	−6.8
		21671525	Hapalindole H	−6.6

SARS-CoV-2: Severe acute respiratory syndrome coronavirus 2; DC-SIGN: Dendritic Cell-Specific Intercellular adhesion molecule-3-Grabbing Non-integrin; AXL: AXL Receptor Tyrosine Kinase; TIM-1: T-cell immunoglobulin and mucin domain 1; ACE2: angiotensin-converting enzyme 2.

**Table 3:** Molecular docking analysis of bacteriocin plantaricin ASM1 (PDB ID: 2MVI) derived from *L. plantarum*.

Receptor		Interacting Amino Acids of Bacteriocin PlantaricinASM1 with Receptor	Interacting Amino Acids of Receptor (Viral Protein)	Active Binding Site/Interacting Domain of Receptor (Viral Protein)	Docking Energy (kcal/mol)
PDB ID	Name				
1TG8	Dengue virus envelope protein	TYR 16	HIS 346	N-Terminal of Domain 1	−990.5
5JQ3	Ebola virus glycoprotein	GLY 39, HIS 42	LYS 588, ASP 591, LEU 594	N-Domain	−1090.6
5JHM	Zika virus protein E	TRP 6, LEU 9, ALA 10, ASP 17	ASP 98, ASN 103, LYS 251, ARG 252	C-Terminal of Domain 1	−1167.4
4QWO	A42R Profilin-like protein of monkeypox virus	LYS 1, ASP 17, TYR 23, TRP 6	ARG 115, ARG 114, SER 73, ASP 76, GLU 83	-	−1102.8
7LWS	Spike (S) protein of SARS-CoV-2 B.1.1.7 UK variant	LYS 1, TRP 4, ALA 14, GLY 15, TYR 16, THR 20, ASP 22, TYR 25, HIS 27, VAL 31, SER 40, HIS 42	LYS 378, ASP 405, ARG 408, PRO 412, ASP 985, GLU 988, ARG 995, LYS 378, TYR 380, GLY 413, VAL 991, GLN 992, ARG 995	N-Terminal of S1 Domain	−1329.9
7LYK	Spike (S) protein of SARS-CoV-2 B.1.351 South African variant	LYS 1, TRP 4, TRP 6, TYR 7, THR 8, THR 20, ASP 22, TYR 23, SER 34, SER 35, GLY 36, SER 40, TYR 41	THR 553, ASP 574, ASP 586, ILE 587, PRO 589, LYS 278, GLU 281, LEU 303, LYS 304, THR 732, VAL 826, ASN 960	The intersection between C-Terminal of S1 Domain and S2 Domain	−1399.3
7M8K	Spike (S) protein of SARS-CoV-2 P.1 Japan/Brazil variant	TRP 6, TYR 7, ASP 17, SER 26, GLY 38, GLY 39	ALA 67, HIS 69, ASP 80, THR 95, GLU 96, PHE 186, ARG 246	C-Terminal of S1 Domain	−1274
7N8H	Spike (S) protein of SARS-CoV-2 B.1.427 USA variant	ALA 10, TYR 16, SER 40, TYR 41, HIS 42	ARG 19, ASP 54, SER 735, VAL 736, ASM 764, THR 768, THR 859	The intersection between N-Terminal of S1 Domain and S2 Domain	−1351
7V7O	Spike (S) protein of SARS-CoV-2 B.1.617.2 Indian variant	LYS 1, TRP 6, THR 8, SER 26, SER 40	THR 825, LEU 826, LYS 852, ASP 865, GLN 947	S2 Domain	−1322.6
7T9J	Spike (S) protein of SARS-CoV-2 B.1.1.529 Omicron variant	LYS 1, TRP 4, TRP 6, SER 18, ASP 22, TYR 23, TYR 25, HIS 27, SER 34, SER 40, TYR 41, HIS 42, CYS 43	GLU 281, SER 305, PHE 306, GLU 309, THR 732, ARG 815, VAL 826, LYS 856, VAL 860, LEU 948, ASN 960, HIS 961	S2 Domain	−1237.5

SARS-CoV-2: Severe acute respiratory syndrome coronavirus 2, TRP: Tryptophan, LEU: Leucine, ALA: Alanine, ASP: Aspartic acid, ASN: Asparagine, LYS: Lysine, ARG: Arginine, GLY: Glutamic acid, PRO: Proline, VAL: Valine, GLU: Glutamic acid, SER: Serine, THR: Threonine, HIS: Histidine, ILE: Isoleucine, CYS: Cysteine, MET: Methionine, PHE: Phenylalanine, TYR: Tyrosine.

**Table 4:** Molecular docking analysis of bacteriocin lactococcin G (PDB ID: 2JPK) derived from *L. lactis*.

Receptor		Interacting Amino Acids of Bacteriocin Lactococcin G with Receptor	Interacting Amino Acids of Receptor (Viral Protein)	Active Binding Site/Interacting Domain of Receptor (Viral Protein)	Docking Energy (kcal/mol)
PDB ID	Name				
1TG8	Dengue virus envelope protein	TRP 8	TRP 212	N-Terminal of Domain 1	−932.5
5JQ3	Ebola virus glycoprotein	LYS 2, TRP 3, ASN 28	THR 600, TRP 615, THR 616	N-Domain	−1056.6
5JHM	Zika virus protein E	LYS 1, ASP 10	ASN 103, ASP 247	N-Terminal of Domain 1	−982.4
4QWO	A42R Profilin-like protein of monkeypox virus	ALA 7, TRP 5, GLU 26, ASN 28, ASP 30, LYS 29, TRP 5, ALA 7	LYS 59, ASN 54, ARG 114, VAL 91, THR 111	-	−880.6
7LWS	Spike (S) protein of SARS-CoV-2 B.1.1.7 UK variant	LYS 1, GLY 4, TRP 5, LEU 6, ASP 10, GLU 14, GLY 20, LYS 29, ASP 30, LYS 33, ASN 34	LYS 1086, ARG 1090, ARG 1091, GLU 1092, HIS 1101, TRP 1102, VAL 1104, ASN 1135, THR 1136, GLN 1142, PRO 1143, GLU 1144	S2 Domain	−1100.1
7LYK	Spike (S) protein of SARS-CoV-2 B.1.351 South African variant	ASP 10, LYS 21, GLU 26, ASN 28, LYS 31, LYS 33, ASN 34	HIS 519, ASN 544, ASP 88, ASP 198, ARG 983	C-Terminal of S1 Domain	−1075.9
7M8K	Spike (S) protein of SARS-CoV-2 P.1 Japan/Brazil variant	LYS 2, ASP 10, GLU 14, GLY 18, LYS 21, GLU 26	THR 95, SER 98, VAL 213, ARG 214, HIS 245, ARG 246, TYR 248	C-Terminal of S1 Domain	−1121.8
7N8H	Spike (S) protein of SARS-CoV-2 B.1.427 USA variant	LYS 1, TRP 5, LEU 6, ASP 10, GLU 14, LYS 17, LYS 21, LYS 25, GLU 26, LYS 29, ASP 30, LYS 31, LYS 33, ASN 34	GLN 644, ASN 334, ARG 357, PRO 463, GLU 465, ARG 466, HIS 519, ASP 40, THR 51, GLN 52, ASP 88, LYS 195, ASP 198, ASN 57	C-Terminal of S1 Domain	−1262.3
7V7O	Spike (S) protein of SARS-CoV-2 B.1.617.2 Indian variant	LYS 1, LYS 2, TRP 3, ASP 10, LYS 21, ASN 34	LYS 41, TYR 168, SER 170, GLN 171, ASN 194, GLY 197, LEU 224, SER 980	N-Terminal of S1 Domain	−1105.7
7T9J	Spike (S) protein of SARS-CoV-2 B.1.1.529 Omicron variant	LYS 1, LEU 6, ASP 10, LYS 21, LYS 33, ASN 34, ILE 35	LYS 41, GLN 115, ASN 165, CYS 166, THR 167, PRO 174, LYS 202, ILE 203, PRO 230, ILE 231, SER 982	N-Terminal of S1 Domain	−1009.9

SARS-CoV-2: Severe acute respiratory syndrome coronavirus 2, TRP: Tryptophan, LEU: Leucine, ALA: Alanine, ASP: Aspartic acid, ASN: Asparagine, LYS: Lysine, ARG: Arginine, GLY: Glutamic acid, PRO: Proline, VAL: Valine, GLU: Glutamic acid, SER: Serine, THR: Threonine, HIS: Histidine, ILE: Isoleucine, CYS: Cysteine, MET: Methionine, PHE: Phenylalanine, TYR: Tyrosine.

**Table 5:** Molecular docking analysis of nisin (PDB ID: 5XHB) derived from *L. lactis*.

Receptor		Interacting Amino Acids of Nisin with Receptor	Interacting Amino Acids of Receptor (Viral Protein)	Active Binding Site/Interacting Domain of Receptor (Viral Protein)	Docking Energy (kcal/mol)
PDB ID	Name				
1TG8	Dengue virus envelope protein	-	-	C-Terminal of Domain 3	−633.2
5JQ3	Ebola virus glycoprotein	TYR 65, LYS 83, ASN 91, PHE 129	THR 600, CYS 601, GLU 611, ASP 614	N-Domain	−737.3
5JHM	Zika virus protein E	ARG 29, ASP 41, ASN 42	HIS 214, GLU 216, TRP 217, ASP 220, GLY 271	C-Terminal of Domain 2	−734.2
4QWO	A42R Profilin-like protein of monkeypox virus	SER 193, ARG 192, GLU 211, ASP 213, ASN 234, ALA 177, LEU 176	PRO 110, THR 112, SER 113, ARG 115, VAL 91, ARG 114, TYR 70	-	−783.6
7LWS	Spike (S) protein of SARS-CoV-2 B.1.1.7 UK variant	LYS 73, LYS 83, ASP 137, ASN 139, PHE 140, VAL 141, ASP 151, ARG 192, SER 193, GLU 194, ASP 213	ALA 27, TYR 28, THR 29, ASN 30, ASN 61, ASP 80, ASN 81, PRO 295, GLU 298, SER 316, GLN 321, THR 599, GLN 607, GLU 619	N-Terminal of S1 Domain	−798.6
7LYK	Spike (S) protein of SARS-CoV-2 B.1.351 South African variant	SER 193, ASP 213, GLU 232	ARG 102, LYS 129, ASN 164, ASN 165	N-Terminal of S1 Domain	−797
7M8K	Spike (S) protein of SARS-CoV-2 P.1 Japan/Brazil variant	AGR 192, SER 193, TRP 195, GLU 211, ASP 213, GLY 215, GLU 216, GLU 232, ASN 234, ASP 235	TRP 64, ASP 80, SER 94, THR 95, LYS 97, PHE 186, HIS 245, ARG 246, LEU 249, THR 250	C-Terminal of S1 Domain	−751.9
7N8H	Spike (S) protein of SARS-CoV-2 B.1.427 USA variant	THR 162, THR 163, VAL 164, ASP 183, SER 186, LYS 188, SER 193, GLU 194, TRP 195, SER 208, ARG 209, GLU 211, ASP 213, ASP 235	ASN 81, VAL 83, GLU 96, LYS 97, ILE 100, ASN 137, ARG 158, LYS 42, LYS 45, THR 56, GLU 1, ASP 110	C-Terminal of S1 Domain	−741.3
7V7O	Spike (S) protein of SARS-CoV-2 B.1.617.2 Indian variant	LYS 50, GLU 55, ILE 142, GLU 171, TYR 172, GLN 173, ASP 174, VAL 175, ALA 177, GLU 178, ARG 180, ARG 192, GLU 211, ASP 213	ARG 401, ASP 403, TYR 447, ARG 450, GLY 480, GLU 482, PHE 488, GLN 491, SER 492, GLN 496, ASN 499, VAL 501, TYR 503, LYS 113, ASN 435, ASN 438, GLN 504	C-Terminal of S1 Domain	−803.6
7T9J	Spike (S) protein of SARS-CoV-2 B.1.1.529 Omicron variant	TYR 152, ASP 166, GLU 167, TYR 172, ASP 174, VAL 175, ALA 177, ASP 183, ARG 192, SER 208, GLU 216, ARG 221	TYR 200, ASP 985, ARG 403, ARG 408, GLU 409, GLY 413, GLN 414, THR 415, ASN 417, TYR 453, LYS 478, TYR 489, ARG 493	C-Terminal of S1 Domain	−826

SARS-CoV-2: Severe acute respiratory syndrome coronavirus 2, TRP: Tryptophan, LEU: Leucine, ALA: Alanine, ASP: Aspartic acid, ASN: Asparagine, LYS: Lysine, ARG: Arginine, GLY: Glutamic acid, PRO: Proline, VAL: Valine, GLU: Glutamic acid, SER: Serine, THR: Threonine, HIS: Histidine, ILE: Isoleucine, CYS: Cysteine, MET: Methionine, PHE: Phenylalanine, TYR: Tyrosine.

**Table 6:** Molecular docking analysis of bacteriocin glycocin F (PDB ID: 2KUY) derived from *L. plantarum*.

Receptor		Interacting Amino Acids of Bacteriocin Glycocin F with Receptor	Interacting Amino Acids of Receptor (Viral Protein)	Active Binding Site/Interacting Domain of Receptor (Viral Protein)	Docking Energy (kcal/mol)
PDB ID	Name				
1TG8	Dengue virus envelope protein	TYR 25	TRP 212	C-Terminal of Domain 2	−1184.6
5JQ3	Ebola virus glycoprotein	GLY 13, TYR 16, SER 38	TRP 597, CYS 609, LYS 622	N-Domain	−1208.2
5JHM	Zika virus protein E	ALA 3, TRP 4, CYS 5, TYR 25, CYS 28	ASP 247, ARG 252, THR 254	N-Terminal of Domain 1	−1015
4QWO	A42R Profilin-like protein of monkeypox virus	ASP 22, LYS 32, SER 36, HIS 33, ASP 17, SER 38, SER 40, TYR 41	ARG 114, ALA 89, ILE 94, LYS 65, GLU 83, PRO 110, THR 112, ARG 115	-	−1144.7
7LWS	Spike (S) protein of SARS-CoV-2 B.1.1.7 UK variant	MET 11, ALA 14, GLY 15, TYR 16, TYR 23, TYR 25, PHE 29, GLY 30, LYS 32	GLU 988, GLN 992, ARG 995, LYS 378, GLN 414, THR 415, ASP 420	N-Terminal of S1 Domain	−1505.6
7LYK	Spike (S) protein of SARS-CoV-2 B.1.351 South African variant	TYR 7, CYS 12, TYR 16, ASP 17, SER 18, THR 20, TYR 23, TYR 25, HIS 27, GLY 30, ILE 31	LYS 386, ASP 389, ASN 544, GLY 545, THR 547, LYS 41, ASP 198, TYR 200, ASP 228, PRO 272, SER 975	C-Terminal of S1 Domain	−1327.4
7M8K	Spike (S) protein of SARS-CoV-2 P.1 Japan/Brazil variant	LYS 1, PRO 2, ALA 3, ALA 10, TYR 16, ASP 17, SER 18, THR 20, TYR 25, CYS 28, PHE 29	TRP 64, HIS 69, ASN 81, GLU 96, LYS 97, ASN 99, ARG 214, ALA 243, HIS 245, ARG 246, SER 247, TYR 248	C-Terminal of S1 Domain	−1364.5
7N8H	Spike (S) protein of SARS-CoV-2 B.1.427 USA variant	LYS 1, ALA 3, TRP 4, MET 11, ASP 17, SER 18, THR 20, ASP 22, TYR 25, IEL 31, LYS 32, HIS 33, HIS 34	LYS 378, TYR 380, PRO 412, GLY 413, GLN 414, GLU 988, GLU 990, HIS 66, LYS 378, TYR 380, GLN 414, ASP 428, TYR 756, ASP 994, ARG 995, THR 998	The intersection between N-Terminal of S1 Domain and C-Terminal of S1 Domain	−1756.7
7V7O	Spike (S) protein of SARS-CoV-2 B.1.617.2 Indian variant	LYS 1, PRO 2, TRP 4, TYR 7, ASP 17, SER 26, HIS 27, CYS 28, PHE 29, LYS 32, HIS 33, HIS 34, SER 35, SER 36, GLY 37, SER 38, SER 39, SER 40, TYR 41, HIS 42, CYS 43	ARG 401, ASP 403, ARG 406, GLN 412, TYR 503, GLU 988, ASP 992, TYR 367, SER 369, PHE 372, SER 373, PHE 375, LYS 376, GLY 402, ASP 425, ASP 426, PHE 427, ASN 435, GLN 990	C-Terminal of S1 Domain	−1333.5
7T9J	Spike (S) protein of SARS-CoV-2 B.1.1.529 Omicron variant	TYP 16, ASP 17, SER 18, THR 20, ASP 22, TYR 25, SER 26, HIS 27, CYS 28, PHE 29, GLY 30, LYS 32, HIS 33, SER 35, SER 36, SER 40	ASN 544, GLU 564, ARG 567, THR 573, ARG 577, LYS 41, HIS 49, GLU 52, ASP 53, GLN 173, ASP 228, LYS 969, SER 975, ARG 983	The intersection between N-Terminal of S1 Domain and C-Terminal of S1 Domain	−1219.4

SARS-CoV-2: Severe acute respiratory syndrome coronavirus 2, TRP: Tryptophan, LEU: Leucine, ALA: Alanine, ASP: Aspartic acid, ASN: Asparagine, LYS: Lysine, ARG: Arginine, GLY: Glutamic acid, PRO: Proline, VAL: Valine, GLU: Glutamic acid, SER: Serine, THR: Threonine, HIS: Histidine, ILE: Isoleucine, CYS: Cysteine, MET: Methionine, PHE: Phenylalanine, TYR: Tyrosine.

**Table 7:** Molecular docking analysis of gardimycin (PDB ID: 1AJ1) derived from *A. garbadinensis*.

Receptor		Interacting Amino Acids of Gardimycinwith Receptor	Interacting Amino Acids of Receptor (Viral Protein)	Active Binding Site/Interacting Domain of Receptor (Viral Protein)	Docking Energy (kcal/mol)
PDB ID	Name				
1TG8	Dengue virus envelope protein	-	-	C-Terminal of Domain 2	−803.7
5JQ3	Ebola virus glycoprotein	ILE 16	TRP 597	N-Domain	−815.6
5JHM	Zika virus protein E	TRP 4	TRP 217	The intersection between N-Terminal of Domain 2 and C-Terminal of Domain 2	−1009
4QWO	A42R Profilin-like protein of monkeypox virus	CYS 17, ALA 18, VAL 15, GLY 13, CYS 12, ALA 18	TYR 118, ARG 114, THR 71	-	−754
7LWS	Spike (S) protein of SARS-CoV-2 B.1.1.7 UK variant	SER 2, GLY 3, VAL 5, CYS 6, LEU 8, CYS 12, ALA 18, CYS 19	THR 588, PRO 589, CYS 590, ASN 616, THR 618, GLU 619, ARG 646	C-Terminal of S1 Domain	−985.8
7LYK	Spike (S) protein of SARS-CoV-2 B.1.351 South African variant	SER 2, GLU 11, GLY 13, VAL 15, IEL 16	ARG 646, HIS 1058	The intersection between C-Terminal of S1 Domain and S2 Domain	−978.5
7M8K	Spike (S) protein of SARS-CoV-2 P.1 Japan/Brazil variant	TRP 4, VAL 5, GLU 11, VAL 15, ILE 16	HIS 69, GLU 96, HIS 245, TYR 248	N-Terminal of S1 Domain	−977.4
7N8H	Spike (S) protein of SARS-CoV-2 B.1.427 USA variant	SER 2, GLY 3, TRP 4, VAL 5, CYS 6, LEU 8, GLU 11, VAL 15, ILE 16, CYS 17, ALA 18	TYR 449, GLY 496, GLU 498, ASN 501, TYR 505, THR 28, PHE 29, TYR 103, ASP 104, TYR 111, ASP 113	N-Terminal of S1 Domain	−998.4
7V7O	Spike (S) protein of SARS-CoV-2 B.1.617.2 Indian variant	GLY 3, LEU 8, VAL 15, ILE 16	THR 732, LEU 826, ASN 958	S2 Domain	−992
7T9J	Spike (S) protein of SARS-CoV-2 B.1.1.529 Omicron variant	GLY 13	ARG 646	S2 Domain	−938.1

SARS-CoV-2: Severe acute respiratory syndrome coronavirus 2, TRP: Tryptophan, LEU: Leucine, ALA: Alanine, ASP: Aspartic acid, ASN: Asparagine, LYS: Lysine, ARG: Arginine, GLY: Glutamic acid, PRO: Proline, VAL: Valine, GLU: Glutamic acid, SER: Serine, THR: Threonine, HIS: Histidine, ILE: Isoleucine, CYS: Cysteine, MET: Methionine, PHE: Phenylalanine, TYR: Tyrosine.

**Table 8:** Molecular docking analysis of surfactin (PDB ID: 1JMK) derived from *B. subtilis*.

Receptor		Interacting Amino Acids of Surfactin with Receptor	Interacting Amino Acids of Receptor (Viral Protein)	Active Binding Site/Interacting Domain of Receptor (Viral Protein)	Docking Energy (kcal/mol)
PDB ID	Name				
1TG8	Dengue virus envelope protein	ASP 116, ASP 118, ARG 120, ASP 180, ASP 182, GLU 185	ASP 98, ARG 99, GLY 102, ASN 103, LYS 110, LYS 246	C-Terminal of Domain 3	−742
5JQ3	Ebola virus glycoprotein	ASP 180, ARG 202, THR 194, THR 195	ASP 614, TRP 615, ASN 618, LYS 622	N-Domain	−952.4
5JHM	Zika virus protein E	ARG 57, SER 115, SER 124, GLU 127, LYS 149, HIS 153, ASN 161, ASP 180, PHE 181, ASP 182	PRO 75, THR 76, GLN 77, GLY 77, GLU 78, LEU 107, PHE 108, THR 313, PHE 314, GLU 320, GLN 331, HIS 401	The intersection between C-Terminal of Domain 3 and N-Terminal of Domain 2	−851.4
4QWO	A42R Profilin-like protein of monkeypox virus	LYS 67, GLY 2, GLY 6, ASP 9	ARG 115, THR 71, THR 112	-	−818.3
7LWS	Spike (S) protein of SARS-CoV-2 B.1.1.7 UK variant	GLN 112, ASP 116, LEU 117, GLY 119, ARG 120, VAL 122, GLU 123, SER 124, ASN 161, GLU 185, TRP 186	CYS 336, SER 366, ASN 370, ASN 388, LYS 529, LYS 417, ARG 457, TYR 473, SER 477	The intersection between C-Terminal of S1 Domain and S2 Domain	−888.1
7LYK	Spike (S) protein of SARS-CoV-2 B.1.351 South African variant	SER 124, ASP 180, PHE 181, ASP 182, TRP 186, ARG 202, GLN 112, GLU 192	ASN 81, LEU 110, ASP 111, CYS 136, ASN 137, GLY 142, ARG 237, GLN 239, LEU 242	N-Terminal of S1 Domain	−884.5
7M8K	Spike (S) protein of SARS-CoV-2 P.1 Japan/Brazil variant	GLY 112, SER 124, ASP 125, TYR 159, GLU 191, ARG 202	ILE 68, HIS 69, VAL 143, ALA 243, HIS 245, ARG 246, SER 247, TYR 248	C-Terminal of S1 Domain	−975.5
7N8H	Spike (S) protein of SARS-CoV-2 B.1.427 USA variant	LYS 149, TYR 156, ASN 161, ASP 180, ASP 182, ILE 183, GLU 185, TRP 186, LEU 187	TRP 64, HIS 66, GLU 96, LYS 97, ASN 99, ILE 100, ARG 158, LYS 187, ARG 214, GLN 3	N-Terminal of S1 Domain	−895.1
7V7O	Spike (S) protein of SARS-CoV-2 B.1.617.2 Indian variant	ASP 107, TYR 109, SER 115, SER 124, ASP 125, GLU 127, ALA 179, ASP 180, PHE 181, ASP 182, GLU 191	ASN 17, LEU 18, ARG 19, ARG 21, THR 22, GLU 23, PRO 25, HIS 66, HIS 243, ARG 244, SER 245	C-Terminal of S1 Domain	−941.2
7T9J	Spike (S) protein of SARS-CoV-2 B.1.1.529 Omicron variant	ASP 116, ASP 118, ASP 180, PHE 181, ASP 182, GLU 185	ARG 346, ASN 439, LYS 440, LYS 444, ASN 448, ASN 450	C-Terminal of S1 Domain	−785.4

SARS-CoV-2: Severe acute respiratory syndrome coronavirus 2, TRP: Tryptophan, LEU: Leucine, ALA: Alanine, ASP: Aspartic acid, ASN: Asparagine, LYS: Lysine, ARG: Arginine, GLY: Glutamic acid, PRO: Proline, VAL: Valine, GLU: Glutamic acid, SER: Serine, THR: Threonine, HIS: Histidine, ILE: Isoleucine, CYS: Cysteine, MET: Methionine, PHE: Phenylalanine, TYR: Tyrosine.

**Table 9:** Molecular docking analysis of silvestrol (CID: 11787114) derived from *Aglaia* spp.

Receptor		Interacting Amino Acids of Receptor	Active Binding Site/Interacting Domain of Receptor	Binding Affinity (kcal/mol)
PDB ID	Name			
1TG8	Dengue virus envelope protein	LYS 58, ASN 124, LYS 202	N-Terminal of Domain 2	−6.2
5JQ3	Ebola virus glycoprotein	THR 294, PHE 290, THR 293	N-Domain	−7.2
5JHM	Zika virus protein E	TRP 217	The intersection between C-Terminal of Domain 2 and N-Terminal of Domain 2	−7.8
4QWO	A42R Profilin-like protein of monkeypox virus	ASN 78, ASN 116, ARG 119, ARG 129	-	−8.2
7LWS	Spike (S) protein of SARS-CoV-2 B.1.1.7 UK variant	GLN 954, ARG 955, ARG 765	S2 Domain	−8
7LYK	Spike (S) protein of SARS-CoV-2 B.1.351 South African variant	GLN 954	The intersection between C-Terminal of S1 Domain and S2 Domain	−8.4
7M8K	Spike (S) protein of SARS-CoV-2 P.1 Japan/Brazil variant	GLN 954, GLN 957	The intersection between C-Terminal of S1 Domain and S2 Domain	−7.6
7N8H	Spike (S) protein of SARS-CoV-2 B.1.427 USA variant	THR 998, GLN 1002	The intersection between C-Terminal of S1 Domain and S2 Domain	−8.2
7V7O	Spike (S) protein of SARS-CoV-2 B.1.617.2 Indian variant	ARG 1012	S2 Domain	−7.2
7T9J	Spike (S) protein of SARS-CoV-2 B.1.1.529 Omicron variant	HIS 954, GLU 1017, ARG 1019, ASN 1023	S2 Domain	−8

SARS-CoV-2: Severe acute respiratory syndrome coronavirus 2, TRP: Tryptophan, LEU: Leucine, ALA: Alanine, ASP: Aspartic acid, ASN: Asparagine, LYS: Lysine, ARG: Arginine, GLY: Glutamic acid, PRO: Proline, VAL: Valine, GLU: Glutamic acid, SER: Serine, THR: Threonine, HIS: Histidine, ILE: Isoleucine, CYS: Cysteine, MET: Methionine, PHE: Phenylalanine, TYR: Tyrosine.

**Table 10:** Molecular docking analysis of andrographolide (CID: 5318517) derived from *A. paniculata*.

Receptor		Interacting Amino Acids of Receptor	Active Binding Site/Interacting Domain of Receptor	Binding Affinity (kcal/mol)
PDB ID	Name			
1TG8	Dengue virus envelope protein	ALA 313, GLU 314, NDG 402	C-Terminal of Domain 2	−6.5
5JQ3	Ebola virus glycoprotein	GLN 570, ARG 574	C-Domain	−7.6
5JHM	Zika virus protein E	LYS 209, HIS 210	The intersection between C-Terminal of Domain 2 and N-Terminal of Domain 2	−6.6
4QWO	A42R Profilin-like protein of monkeypox virus	ASN 14, TYR 80, ARG 127, ARG 129	-	−7.4
7LWS	Spike (S) protein of SARS-CoV-2 B.1.1.7 UK variant	GLN 1002, TYR 756, TYR 756, ASP 994, THR 998	N-Terminal of S1 Domain and C-Terminal of S1 Domain	−7.7
7LYK	Spike (S) protein of SARS-CoV-2 B.1.351 South African variant	ARG 1014, ARG 1019	S2 Domain	−7.5
7M8K	Spike (S) protein of SARS-CoV-2 P.1 Japan/Brazil variant	ARG 1014	S2 Domain	−7.6
7N8H	Spike (S) protein of SARS-CoV-2 B.1.427 USA variant	PHE 970	The intersection between C-Terminal of S1 Domain and S2 Domain	−7
7V7O	Spike (S) protein of SARS-CoV-2 B.1.617.2 Indian variant	GLN 1000	The intersection between C-Terminal of S1 Domain and S2 Domain	−7.7
7T9J	Spike (S) protein of SARS-CoV-2 B.1.1.529 Omicron variant	THR 961, SER 758, ARG 765	The intersection between N-Terminal of S1 Domain and S2 Domain	−6.9

SARS-CoV-2: Severe acute respiratory syndrome coronavirus 2, TRP: Tryptophan, LEU: Leucine, ALA: Alanine, ASP: Aspartic acid, ASN: Asparagine, LYS: Lysine, ARG: Arginine, GLY: Glutamic acid, PRO: Proline, VAL: Valine, GLU: Glutamic acid, SER: Serine, THR: Threonine, HIS: Histidine, ILE: Isoleucine, CYS: Cysteine, MET: Methionine, PHE: Phenylalanine, TYR: Tyrosine.

**Table 11:** Molecular docking analysis of lyngbyabellin A (CID:10032587).

Receptor		Interacting Amino Acids of Receptor	Active Binding Site/Interacting Domain of Receptor	Binding Affinity (kcal/mol)
PDB ID	Name			
1TG8	Dengue virus envelope protein	GLY 275, LYS 128	N-Terminal of Domain 2	−5.8
5JQ3	Ebola virus glycoprotein	GLU 156, LYS 84, SER 81	N-Domain	−7.2
5JHM	Zika virus protein E	THR 267	The intersection between C-Terminal of Domain 2 and N-Terminal of Domain 2	−6.2
4QWO	A42R Profilin-like protein of monkeypox virus	ASN 78, ARG 127, ARG 129	-	−8.7
7LWS	Spike (S) protein of SARS-CoV-2 B.1.1.7 UK variant	ARG 1019, AGR 1014	S2 Domain	−9
7LYK	Spike (S) protein of SARS-CoV-2 B.1.351 South African variant	THR 739, ASN 317	The intersection between C-Terminal of S1 Domain and S2 Domain	−7.5
7M8K	Spike (S) protein of SARS-CoV-2 P.1 Japan/Brazil variant	GLN 954, AGR 765	The intersection between C-Terminal of S1 Domain and S2 Domain	−8.6
7N8H	Spike (S) protein of SARS-CoV-2 B.1.427 USA variant	THR 998, TYR 756	The intersection between C-Terminal of S1 Domain and S2 Domain	−8
7V7O	Spike (S) protein of SARS-CoV-2 B.1.617.2 Indian variant	ARG 1012	S2 Domain	−8.5
7T9J	Spike (S) protein of SARS-CoV-2 B.1.1.529 Omicron variant	TYR 313	S2 Domain	−8

SARS-CoV-2: Severe acute respiratory syndrome coronavirus 2, TRP: Tryptophan, LEU: Leucine, ALA: Alanine, ASP: Aspartic acid, ASN: Asparagine, LYS: Lysine, ARG: Arginine, GLY: Glutamic acid, PRO: Proline, VAL: Valine, GLU: Glutamic acid, SER: Serine, THR: Threonine, HIS: Histidine, ILE: Isoleucine, CYS: Cysteine, MET: Methionine, PHE: Phenylalanine, TYR: Tyrosine.

**Table 12:** Molecular docking analysis of hapalindole H (CID:21671525).

Receptor		Interacting Amino Acids of Receptor	Active Binding Site/Interacting Domain of Receptor	Binding Affinity (kcal/mol)
PDB ID	Name			
1TG8	Dengue virus envelope protein	GLN 200, LYS 128, LEU 277, ALA 50	The intersection between C-Terminal of Domain 2 and N-Terminal of Domain 2	−6.5
5JQ3	Ebola virus glycoprotein	ARG 89, LYS 155	N-Domain	−7.8
5JHM	Zika virus protein E	THR 267	The intersection between C-Terminal of Domain 2 and N-Terminal of Domain 2	−7.2
4QWO	A42R Profilin-like protein of monkeypox virus	ASN 14, ASN 78, TYR 80, HIS 100, ASP 100, ARG 127, ARG 129	-	−8.1
7LWS	Spike (S) protein of SARS-CoV-2 B.1.1.7 UK variant	GLN 965	The intersection between N-Terminal of S1 Domain and C-Terminal of S1 Domain	−7.6
7LYK	Spike (S) protein of SARS-CoV-2 B.1.351 South African variant	ARG 765, GLN 954	S2 Domain	−8.2
7M8K	Spike (S) protein of SARS-CoV-2 P.1 Japan/Brazil variant	THR 768, SER 735, ASN 764	The intersection between C-Terminal of S1 Domain and S2 Domain	−8.2
7N8H	Spike (S) protein of SARS-CoV-2 B.1.427 USA variant	VAL 382, GLY 381	The intersection between N-Terminal of S1 Domain and C-Terminal of S1 Domain	−7.2
7V7O	Spike (S) protein of SARS-CoV-2 B.1.617.2 Indian variant	ILE 768	S2 Domain	−7.4
7T9J	Spike (S) protein of SARS-CoV-2 B.1.1.529 Omicron variant	ARG 1014	The intersection between C-Terminal of S1 Domain and S2 Domain	−8

SARS-CoV-2: Severe acute respiratory syndrome coronavirus 2, TRP: Tryptophan, LEU: Leucine, ALA: Alanine, ASP: Aspartic acid, ASN: Asparagine, LYS: Lysine, ARG: Arginine, GLY: Glutamic acid, PRO: Proline, VAL: Valine, GLU: Glutamic acid, SER: Serine, THR: Threonine, HIS: Histidine, ILE: Isoleucine, CYS: Cysteine, MET: Methionine, PHE: Phenylalanine, TYR: Tyrosine.

**Table 13:** Comparison of molecular docking analysis of mutant bacterial AMPs with corresponding wild-type proteins.

Target Viral Receptor		Bacterial AMP		Wild-Type AMP		Mutant AMP	
				Docking Energy (kcal/mol)	Amino Acid Residue/s	Docking Energy (kcal/mol)	Amino Acid Residue/s
Name	PDB Code	Name	PDB Code				
Spike (S) protein of SARS-CoV-2 B.1.1.7 UK variant	7LWS	Bacteriocin glycocin F	2KUY	−1505.6	MET 11, ALA 14, GLY 15, TYR 16, TYR 23, TYR 25, PHE 29, GLY 30, LYS 32	−1344.8	LEU 11, PHE 14, THR 15, VAL 16, VAL 23, TYR 25, VAL 29, PRO 30, PRO 32
Spike (S) protein of SARS-CoV-2 B.1.427 USA variant	7N8H	Bacteriocin glycocin F	2KUY	−1756.7	LYS 1, ALA 3, TRP 4, MET 11, ASP 17, SER 18, THR 20, ASP 22, TYR 25, IEL 31, LYS 32, HIS 33, HIS 34	−1431.1	VAL 1, SER 3, PRO 4, LEU 11, CYS 17, VAL 18, PRO 20, ASP 22, VAL 25, PRO 31, LEU 32, PRO 33, THR 34
Spike (S) protein of SARS-CoV-2 B.1.617.2 Indian variant	7V7O	Bacteriocin glycocin F	2KUY	−1333.5	LYS 1, TRP 4, ASP 17, SER 26, HIS 27, CYS 28, PHE 29, LYS 32, HIS 33, HIS 34, SER 35, SER 36, GLY 37, SER 38, HIS 42	−1468.3	VAL 1, PRO 4, CYS 17, VAL 26, VAL 27, LEU 28, SER 29, PRO 32, PRO 33, THR 34, VAL 35, VAL 36, VAL 37, VAL 38,PRO 42
Spike (S) protein of SARS-CoV-2 P.1 Japan/Brazil variant	7M8K	Bacteriocin glycocin F	2KUY	−1364.5	LYS 1, PRO 2, ALA 3, ALA 10, TYR 16, ASP 17, SER 18, THR 20, TYR 25, CYS 28, PHE 29	−1473.0	VAL 1, VAL 2, PRO 3, VAL 10, VAL 16, PRO 17, VAL 18, VAL 20, VAL 25, PRO 28, VAL 29
Spike (S) protein of SARS-CoV-2 B.1.351 South African variant	7LYK	Bacteriocin plantaricin ASM1	2MVI	−1399.3	LYS 1, TRP 4, TRP 6, TYR 7, THR 8, THR 20, ASP 22, TYR 23, SER 34, SER 35, GLY 36, SER 40, TYR 41,	−1228.8	LEU 1, CYS 4, CYS 6, THR 7, THR 8, CYS 20, CYS 22, VAL 23, VAL 34, PRO 35, PRO 36, PRO 40, PRO 41
Spike (S) protein of SARS-CoV-2 B.1.1.529 Omicron variant	7T9J	Bacteriocin plantaricin ASM1	2MVI	−1237.5	LYS 1, TRP 4, TRP 6, SER 18, ASP 22, TYR 23, TYR 25, HIS 27, SER 34, SER 40, TYR 41, HIS 42, CYS 43	−1002.0	LEU 1, CYS 4, CYS 6, LEU 18, CYS 22, VAL 23, THR 25, PRO 27, PRO 34, PRO 40, PRO 41, PRO 42, CYS 43
Dengue virus envelope protein	1TG8	Bacteriocin glycocin F	2KUY	−1184.6	TYR 25	−1267.7	VAL 25
Ebola virus glycoprotein	5JQ3	Bacteriocin glycocin F	2KUY	−1208.2	GLY 13, TYR 16, SER 38	−1158.3	VAL 13, PRO 16, VAL 38
Zika virus protein E	5JHM	Bacteriocin plantaricin ASM1	2MVI	−1167.4	TRP 6, LEU 9, ALA 10, ASP 17	−1066.3	CYS 6, VAL 9, PRO 10, PRO 17
A42R Profilin-like protein of monkeypox virus	4QWO	Bacteriocin glycocin F	2KUY	−1144.7	ASP 22, LYS 32, SER 36, HIS 33, ASP 17, SER 38, SER 40, TYR 41	−1054.6	TYR 22, PRO 32, TYR 36, PRO 33, PHE 17, VAL 38, SER 40, VAL 41

The AMPs with the highest affinities for the viral proteins were mutated and the mutants were then evaluated for their affinity for the viral proteins. The increased binding affinities of the mutants are indicated in bold. SARS-CoV-2: Severe acute respiratory syndrome coronavirus 2, TRP: Tryptophan, LEU: Leucine, ALA: Alanine, ASP: Aspartic acid, ASN: Asparagine, LYS: Lysine, ARG: Arginine, GLY: Glutamic acid, PRO: Proline, VAL: Valine, GLU: Glutamic acid, SER: Serine, THR: Threonine, HIS: Histidine, ILE: Isoleucine, CYS: Cysteine, MET: Methionine, PHE: Phenylalanine, TYR: Tyrosine.

## Data Availability

Not applicable.

## Supplementary Materials

The following supporting information can be downloaded at: https://www.mdpi.com/article/10.3390/ijms231911131/s1. Table S1. Ligands (biomolecules) and cellular virus receptors subjected to molecular docking in the experiment.

## Abbreviations

MERS: Middle east respiratory syndrome; SARS: Severe acute respiratory syndrome; COVID-19: Coronavirus disease 2019; SARS-CoV-2: Severe acute respiratory syndrome coronavirus 2; AMPs: antimicrobial peptides; PDB: Protein Data Bank; RCSB: Research Collaboratory for Structural Bioinformatics; SDF spatial data file; NLM: National Library of Medicine; *A. paniculata*: *Andrographis paniculata*; Å: Angstrom; RMSD: Root mean square deviation; DENV: Dengue virus; EBOV: Ebola virus; ZIKV: Zika virus; MPV: Monkeypox virus; HCoV: Human coronavirus; RNA: Ribonucleic acid; CHIKV: Chikungunya virus; HEV: Hepatitis E virus; *L. lactis*; *L. plantarum*: *Lactobacillus plantarum*; *B. subtilis*: *Bacillus subtilis*; HIV: Human immunodeficiency virus; BVDV: Bovine viral diarrhea virus; FDA: The United States Food and Drug Administration; PEDV: Porcine epidemic diarrhea virus; TGEV: Transmissible gastroenteritis coronavirus; μg/mL: microgram per milliliters; nM: nanomoles; kcal/mol: kilocalorie per mole; DC-SIGN: Dendritic Cell-Specific Intercellular adhesion molecule-3-Grabbing Non-integrin; AXL: AXL Receptor Tyrosine Kinase; TIM-1: T-cell immunoglobulin and mucin domain 1; ACE2: angiotensin converting enzyme 2; TRP: Tryptophan, LEU: Leucine; ALA: Alanine; ASP: Aspartic acid; ASN: Asparagine; LYS: Lysine; ARG: Arginine; GLY: Glutamic acid; PRO: Proline; VAL: Valine; GLU: Glutamic acid; SER: Serine; THR: Threonine; HIS: Histidine, ILE: Isoleucine; CYS: Cysteine; MET: Methionine; PHE: Phenylalanine; TYR: Tyrosine.
