# Supplementary material for: Molecular Docking and In-Silico Analysis of Natural Biomolecules against Dengue, Ebola, Zika, SARS-CoV-2 Variants of Concern and Monkeypox Virus
Source: Int J Mol Sci. 2022 Sep 22;23(19):11131. doi: 10.3390/ijms231911131 (PMC9569982; doi:10.3390/ijms231911131)
Supplement: Supplementary file 1 [file ijms-23-11131-s001.zip › ijms-1876888-supplementary.pdf]

Table S1. Ligands (biomolecules) and cellular virus receptors subjected to molecular docking in the experiment.

Ligand		Source	Molecule type
**PDB ID**	**Name**		
2MVI	Bacteriocin plantaricin	*Lactobacillus plantarum*	Bacterial AMP
	ASM1	*Lactobacillus plantarum*	Bacterial AMP
2KUY	Bacteriocin glycocin F		Bacterial AMP
2JPK	Bacteriocin lactococcin	*Lactococcus lactis*	Bacterial AMP
	G	*Lactococcus lactis*	Bacterial AMP
5XHB	Nisin		Bacterial AMP
1AJ1	Gardimycin	*Actinoplanesgar badinensis*	
1JMK	Surfactin	*Bacillus subtilis*	
2R69	Fab 1A1D-2		DENV neutralizing antibody
5JHL	2A10G6 Fab		ZIKV neutralizing antibody
5FHB	mAb100	Human immune system	EBOV neutralizing antibody
7JMX	COVA1-16 Fab		SARS-CoV-2 neutralizing antibody
1SL4	DC-SIGN	Mammalian immune system dendritic cells	Cell antigen receptor for DENV
5U6B	AXL	Mammalian bone marrow stroma and myeloid cells	Cell antigen receptor for ZIKV
2OR8	TIM-1	Mammalian immune system T cells	Cell antigen receptor for EBOV
7JMX	ACE2	Mammalian system cells	Cell antigen receptor for SARS-CoV-2
3J0A	Toll-like receptor 5	Mammalian system cells	Hypothetical cell antigen receptor for MPV
4M76	CR3/Mac-1		

5LGD	CD36		
1H9V	FcγRIIA		
**Conformer ID**			
145996610	Molnupiravir	-<br>-	Anti-SARS-cov-2 drug candidate
121304016	Remdesivir	-	Anti-EBOV drug candidate
45375808	Sofosbuvir	-	Anti-DENV and anti-ZIKV drug candidate
483477	Brincidofovir		Antiviral drug for human MPV
16124688	Tecovirimat	-	Antiviral drug for human MPV
11787114	Silvestrol	*Aglaia* spp.	Phytochemical compound
5318517	Andrographolide	*Andrographis paniculata*	Phytochemical compound
10032587	Lyngbyabellin A	Cyanobacteria	Bacterial secondary metabolite
21671525	Hapalindole H	Cyanobacteria	Bacterial secondary metabolite

SARS-CoV-2: Severe acute respiratory syndrome coronavirus 2; DENV: Dengue virus; EBOV: Ebola virus; ZIKV: Zika virus; DC-SIGN: Dendritic Cell-Specific Intercellular adhesion molecule-3-Grabbing Non-integrin; AXL: AXL Receptor Tyrosine Kinase; TIM-1: T-cell immunoglobulin and mucin domain 1; ACE2: angiotensin converting enzyme 2; Antimicrobial peptide: AMP
